# 199 EDUcational Course Assessment TOOLkit (EDUCATOOL): development and application in a Health-Enhancing Physical Activity (HEPA) promotion intervention

**DOI:** 10.1093/eurpub/ckae114.164

**Published:** 2024-09-26

**Authors:** Tena Matolić, Danijel Jurakić, Zrinka Greblo Jurakić, Tošo Maršić

**Affiliations:** Faculty of Kinesiology, University of Zagreb, Zagreb, Croatia; Faculty of Kinesiology, University of Zagreb, Zagreb, Croatia; Faculty of Croatian Studies, University of Zagreb, Zagreb, Croatia; Faculty of Kinesiology, Josip Juraj Strossmayer University of Osijek, Osijek, Croatia

## Abstract

**Purpose:**

The evaluation of educational courses on HEPA promotion often lacks an empirically defined evaluation framework. Thus, our primary aim was to develop an assessment toolkit called EDUCATOOL, grounded in Kirkpatrick’s evaluation framework, and to assess its measurement properties. Our second goal was to apply EDUCATOOL to evaluate the Sports Club for Health (SCforH) educational course.

**Methods:**

EDUCATOOL was developed through a rigorous four-stage process: 1) literature review, 2) drafting questionnaires based on 150 topic-related publications and discussion among three researchers, 3) refining the questionnaires through the Delphi method including five multidisciplinary experts, and 4) a consultative process with 20 potential end-users. The toolkit was then utilised to evaluate the SCforH educational course, completed by 840 participants from 34 European countries. Participant types included: a) representative of sports club, b) representative of sports association, c) representative of governmental body, d) representative of public health institute and National Physical Activity Focal Point (“HEPA promotors & researchers”), f) academic staff in a higher education or research institution, g) higher education student, and h) other.

**Results:**

EDUCATOOL consists of a post-course questionnaire, follow-up questionnaire, user manual, and data processing calculator. Both questionnaires demonstrated a good fit for Kirkpatrick’s 4-factor model, encompassing Reaction, Learning, Behavioural intent (post-course)/Behaviour (follow-up), and Expected outcomes (post-course)/Results (follow-up). Internal consistency, test-retest reliability factorial validity, and convergent validity were found to be adequate for both questionaries. The SCforH course received high ratings from all participant types, with median scores ranging from 8.00 to 9.00 out of 10 for individual items and from 20.00 to 21.67 out of 25 for each of the four summary components. HEPA promotors and researchers rated the outcome components most highly compared to other participant types.

**Conclusions:**

The EDUCATOOL toolkit represents a valuable resource for evaluating the effectiveness of educational courses and fostering evidence-based practices in various HEPA promotion interventions. Findings from the SCforH course evaluation indicate that innovative and engaging methods in HEPA promotion are well-received by various types of stakeholders.

**Support/Funding Source:**

The study was funded by Erasmus+ Collaborative Partnerships grant (ref: 613434-EPP-1-2019-1-HR9 SPO-SCP) and Croatian Science Foundation (ref: DOK-2020-01-8078).

